# Avulsion fracture of the anterior superior iliac spine: misdiagnosis of a bone tumour

**DOI:** 10.1007/s10195-011-0153-z

**Published:** 2011-08-12

**Authors:** B. S. Dhinsa, Azal Jalgaonkar, Bhupinder Mann, Sajid Butt, Rob Pollock

**Affiliations:** 1 Royal National Orthopaedic Hospital, Stanmore, Middlesex HA7 4LP UK; 2 16 Yeoman Drive, Darland View, Gillingham, Kent ME7 3EL UK

**Keywords:** Avulsion fracture, Anterior superior iliac spine, Adolescent apophyseal injury

## Abstract

Avulsion fractures of the anterior superior iliac spine are rare. This injury is usually seen in adolescents, as an avulsion fracture of the apophyses, a result of sudden vigorous contraction or repetitive contraction of the sartorius and tensor fasciae latae muscles. Treatment for this injury is usually conservative; however, surgical management has been reported in those with significant displacement. We present a 14 year old male patient who was referred to our unit for biopsy of a possible pathological fracture of his right ilium. The authors feel it is essential to understand the importance of ruling out a bone tumour, if the possibility has been raised, before managing a suspected fracture. If there is any doubt, the case should be referred to an appropriate sarcoma unit for review prior to any intervention.

## Introduction

Avulsion fractures of the pelvis are rare injuries that occur when a tendon or ligament contracts suddenly and forcefully to remove a part of the bone it is attached to. They are most commonly seen affecting the growing apophyses of adolescents, and are often missed on initial presentation.

An apophysis, an outgrowth, can be defined as a growth centre where a tendon attaches to bone. As would be expected, its cartilaginous growth plate remains weaker than the attached musculotendinous unit until it fuses at the time of skeletal maturation, and thus is more prone to fracture when the musculotendinous unit is suddenly and vigorously contracted [1]. Adolescents involved in athletic pursuits such as football, sprinting and gymnastics are more likely to suffer from apophyseal avulsion injuries due to the sudden powerful muscular contractions that are required in these activities, as well as the abrupt directional changes of motion performed [2].

The anterior superior iliac spine, which develops from an anterior apophysis of the iliac crest, is the site of origin of the sartorius muscle and part of the tensor fasciae latae. Therefore, an avulsion fracture of the anterior superior iliac spine is most commonly due to forceful contraction or sudden repetitive actions of these muscles, as occurs when running or kicking a ball [3, 4].

The treatment of choice for this injury remains conservative: rest, analgesia, anti-inflammatories and rehabilitation; however, surgical treatment has been utilised depending on the degree of fracture displacement and the individual’s rehabilitation requirements [5, 6].

## Case report

A 14-year-old male patient presented with a 1 week history of pain in his anterior right thigh and anterior pelvic region, which was exacerbated by walking. This was preceded by an injury sustained whilst playing football. He was kicking a football when a severe pain in his right thigh developed, which rendered him unable to walk, and he had to crawl off the football pitch. On examination his gait was affected by pain in the right thigh, there was tenderness to the anterior superior iliac spine on palpation, and all hip movements were limited by pain.

Plain film radiographs were reported by a local district general hospital radiologist as showing a faint ossification within the soft tissues immediately inferior and adjacent to the right anterior inferior iliac spine, suggestive of a rectus femoris avulsion fracture. However, when reviewed in clinic the consultant orthopaedic surgeon noted that there was some calcification around the anterior superior iliac spine which did not collaborate with the time since injury, and a magnetic resonance imaging (MRI) scan was performed.

**Fig. 1:**
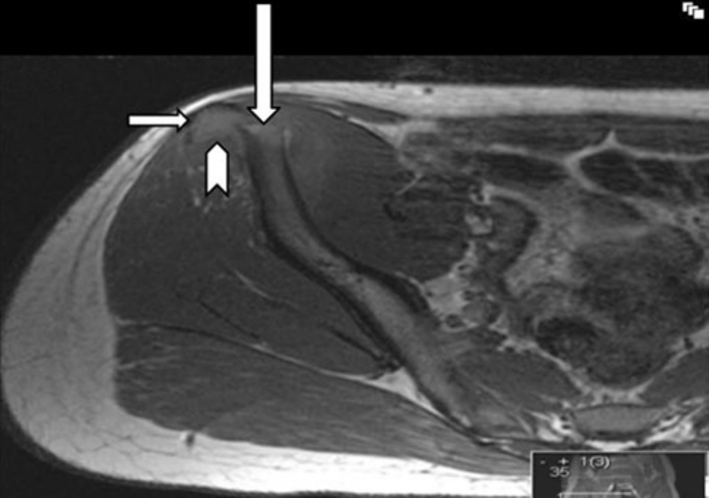
Axial T1-weighted image. Note the sharp anterior superior iliac spine fracture line (*long white arrow*) with retracted sartorius tendon margin (*short white arrow*). Also note that the signal intensity of haematoma is slightly hyperintense (as compared to skeletal muscle, indicated by the *chevron mark*), which is suggestive of a haemorrhagic product rather than a tumour mass

**Fig. 2:**
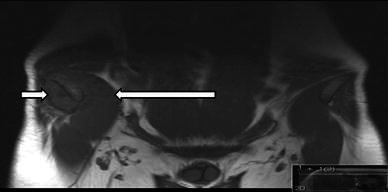
Coronal T1-weighted image. Note the slightly hyperintense haematoma (*short white arrow*). Signal is compared to skeletal muscle (*long white arrow*)

The imaging reported an abnormality in the anterior right iliac crest with an avulsion fracture and a surrounding soft tissue mass (Fig. 1). This solid mass was centred on the anterior superior iliac spine and measured 3 × 4 × 5 cm in size. The signal intensity of this mass was mildly hyperintense on T1-weighted images (Figs. 1a, 2). At the referring hospital, these findings were thought not to correspond to a simple trauma, and suggested a possible underlying bone tumour.

**Fig. 3:**
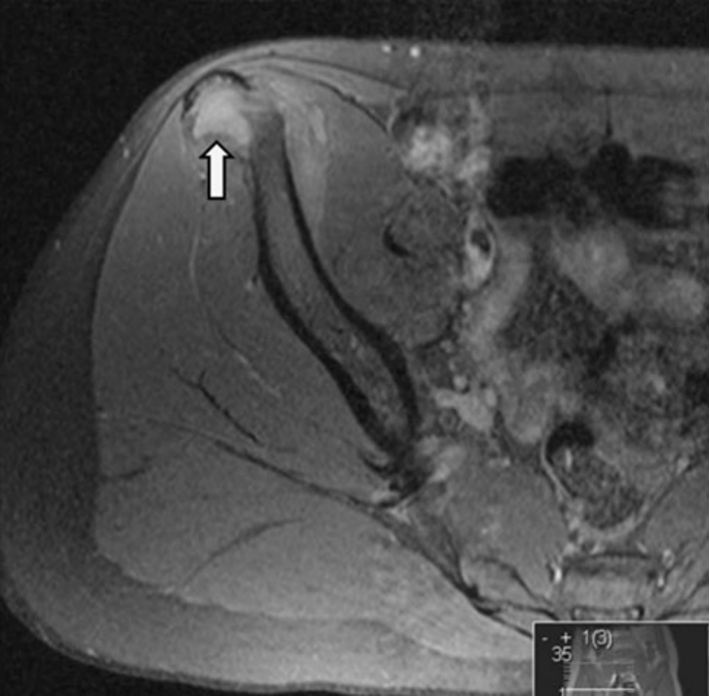
Axial T1 fat-saturated pre-gadolinium image. Note that the surrounding haematoma shows a bright signal indicating haemorrhagic components (*short white arrow*)

The patient was referred to our sarcoma unit for an opinion, where the images were reviewed at a multi-disciplinary meeting. On the review of radiology performed by our specialist musculoskeletal radiologist, it was felt that there was an avulsion fracture of the anterior superior iliac spine with anterolateral displacement of the apophysis and a large surrounding haematoma (Figs. 1, 2, 3). There was mild enhancing marrow oedema present in the anterior third of the iliac blade, but the signal intensity in the bone was otherwise normal. There was nothing to suggest that an infiltrative osseous tumour was present.

**Fig. 4:**
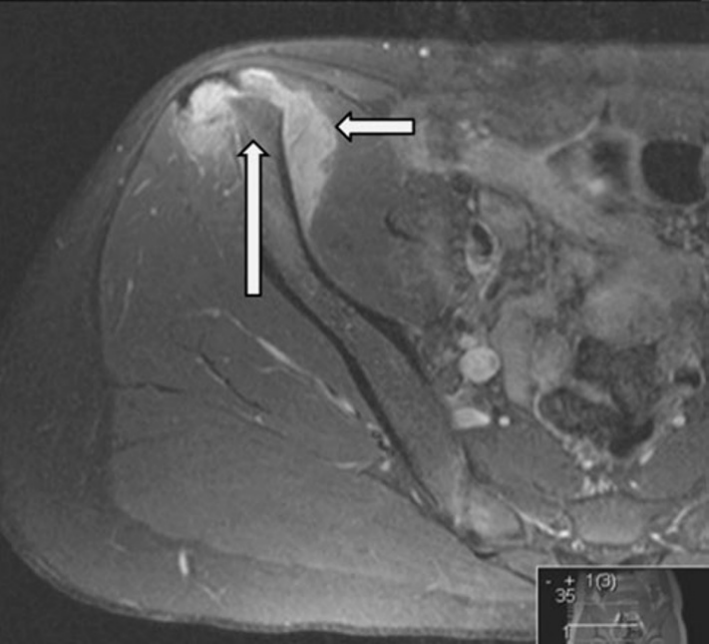
Axial post-intravenous gadolinium T1 fat-saturated image. The haematoma also shows diffuse solid enhancement (*short white arrow*). There is, however, no enhancement in bone (*long white arrow*). Bone tumours with surrounding mass usually show marrow infiltration indicated by low T1 and bright T2 signals in bone that enhance with gadolinium

Radiological features which were supportive of the diagnosis of a fracture rather than a tumour were:

- Presence of a clear fracture with avulsed tendon of sartorius.
- Bone marrow signal in the anterior superior Iliac spine was relatively normal with only a subtle oedema seen.
- The haematoma showed a slightly hyperintense T1 signal intensity and bright T2 fat-saturated (FS) signal. Tumour masses generally show low T1 and bright T2 FS signals (see Fig. 4).

**Fig. 5:**
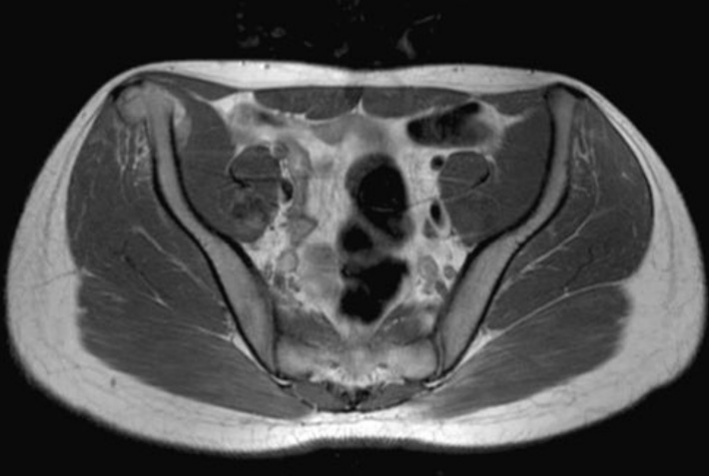
Axial proton density images obtained 3 months after the first scan indicate that the avulsion fracture shows signs of healing. The surrounding haematoma is smaller. Bone marrow signal is also normal

**Fig. 6:**
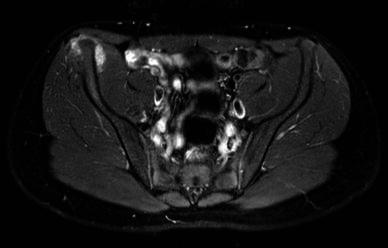
Fat-saturated images obtained 3 months after the first scan show signs of fracture healing

Based upon the clinical history, examination and radiological findings, the MDT decision was that this was an avulsion fracture of the anterior superior iliac spine apophyses. The decision was made to treat conservatively: with rest, analgesia and physiotherapy. A biopsy was not felt necessary at this stage, but a careful, regular clinical follow-up was ensured. An interval MRI scan, performed after 3 months (Fig. 5; see also Fig. 6), showed that the avulsion fracture was demonstrating signs of healing. The initial haematoma was smaller, the degree of marrow oedema was significantly less, and there were no features of bone marrow oedema.

At 1 year follow-up, the patient had normal gait, there was no tenderness of the anterior superior iliac spine on palpation, and he had pain-free full range of movement of the hip. The patient had returned to active sports without any restriction.

## Discussion

Whilst apophyseal avulsion fractures are rarely seen, avulsion fractures of the anterior superior iliac spine are found more commonly in adolescents undergoing vigorous activity. This injury usually occurs as a result of sudden, vigorous or repetitive contraction of the sartorius muscle and tensor fasciae latae. It is sprint athletes, football players and gymnasts that are most often affected, due to the range of movements they perform and sudden changes in direction required.

Diagnosis can be achieved by clinical examination with plain radiograph, and MRI or computed tomography (CT) as determined by availability. Treatment is usually conservative, with rest, analgesia and anti-inflammatories yielding good reported results [7, 8]. Surgical intervention may be required in selected cases when it is desirable to have as short a rehabilitation time as possible, such as in professional athletes, and in those with significantly displaced fractures [5, 6].

These avulsion injuries usually have an associated haematoma and develop excessive callus with post-fracture osteolysis, which on radiological investigation may be mistaken for an osteosarcoma, Ewing’s sarcoma, or an infective process [1]. If the suspicion of a bone tumour has been raised, this must be ruled out before instigating a management plan for the fracture. If there is any doubt the case should be referred to a specialist sarcoma unit for an opinion, ideally in a multi-disciplinary team setting.

Features that support a diagnosis of possible underlying malignancy are relatively trivial traumas resulting in excessive bone injury. On imaging, plain films can show permeative destruction of bone, aggressive new bone formation (indicating osteosarcoma—shown by a sun-ray appearance, Codman’s triangle and cumulus cloud osseous matrix). MR features suggestive of a tumour are poorly defined oedema in bone, surrounding solid soft tissue mass that usually shows low T1 and bright T2 signal intensity mass which is disproportionally large compared to bone involvement (especially in cases of Ewing’s sarcoma and lymphoma).

Infection on plain films shows periosteal reaction, sequestrum and possible lytic lesions. MRI can show necrotic pus with a rim enhancement. Myositis ossificans is soft tissue ossification that develops after trauma and is characterised by a rim of new bone formation. MRI features of this entity can be confused with a soft tissue tumour, especially because there can be surrounding oedema. The typical rim-shaped mineralization is, however, appreciated on careful analysis of MRI and CT scan, and is pathognomonic for myositis ossificans.

The authors feel it is vital that in such cases surgical reduction and fixation should not be performed until an opinion has been sought from a specialist tumour centre. There are helpful signs on various imaging studies which point towards the diagnosis of a fracture vis-à-vis tumour. Careful clinical and radiological follow-up is important to ensure that the initially observed lesion is demonstrating signs of healing.
